# High Sensitivity Differential Giant Magnetoresistance (GMR) Based Sensor for Non-Contacting DC/AC Current Measurement

**DOI:** 10.3390/s20010323

**Published:** 2020-01-06

**Authors:** Cristian Mușuroi, Mihai Oproiu, Marius Volmer, Ioana Firastrau

**Affiliations:** Department of Electrical Engineering and Applied Physics, Transilvania University of Brasov, 29 Blvd. Eroilor, 500036 Brasov, Romaniamihai.oproiu@unitbv.ro (M.O.); firastrau@unitbv.ro (I.F.)

**Keywords:** current sensors, GMR effect, spin-valve sensor, micromagnetic simulations, Bias magnetic field

## Abstract

This paper presents the design and implementation of a high sensitivity giant magnetoresistance (GMR) based current sensor with a broad range of applications. The novelty of our approach consists in using a double differential measurement system, based on commercial GMR sensors, with an adjustable biasing system used to linearize the field response of the system. The work aims to act as a fully-operational proof of concept application, with an emphasis on the mode of operation and methods to improve the sensitivity and linearity of the measurement system. The implemented system has a broad current measurement range from as low as 75 mA in DC and 150 mA in AC up to 4 A by using a single setup. The sensor system is also very low power, consuming only 6.4 mW. Due to the way the sensors are polarized and positioned above the U-shaped conductive band through which the current to be measured is flowing, the differential setup offers a sensitivity of about between 0.0272 to 0.0307 V/A (signal from sensors with no amplifications), a high immunity to external magnetic fields, low hysteresis effects of 40 mA, and a temperature drift of the offset of about −2.59 × 10^−4^ A/°C. The system provides a high flexibility in designing applications where local fields with very low amplitudes must be detected. This setup can be redesigned for a wide range of applications, thus allowing further specific optimizations, which would provide an even greater accuracy and a significantly extended operation range.

## 1. Introduction

Modern electronics applications often require accurate current measurements in a compact design, thus increasing the need for low power current sensing devices. Also, due to the extremely competitive market for power electronics devices, low cost for those current sensing devices is critical.

Contactless current measurement devices are based on detection of the magnetic field created by the current. When only the AC current component is measured, the most used devices are based on current transformers and Rogowski coils [1,2,3]. However, to measure DC/AC currents, sensors able to detect DC magnetic fields with high accuracy must be used.

The (micro)fluxgate [4,5] sensors offer high performance and stability in detection of DC/AC currents. A Fluxgate current sensor uses a high permeability magnetic core to detect magnetic fields produced by a current flow. A system of coils such as the fluxgate coil, driven by a square wave current, compensation coil, and pick-up coil are used to determine the magnetization state of the magnetic core and, hence, the current to be measured. The electronics used to drive the currents, to demodulate the signal and to manage all the sensor’s functionality is quite complex and often power consuming. Now, new reported developments are ongoing, in which the fluxgate magnetometer is co-integrated along with circuitry on a die [5]. Fluxgate sensors are much more sensitive than Hall sensors and have better temperature stability, and low noise and linearity. A main disadvantage is their relatively small full range of operation, of about 2 mT. In [4] 16 integrated microfluxgate sensors TI DRV425 were used, which were placed around an Al conductor with the cross-section of 100 × 10 mm^2^ able to support a current of 400 A. Using this complex system, composed from sensors, DAQ boards, and Mini-PC, a resolution of 1 mA and a temperature drift of 8 mA/°C were achieved for a maximum measured current of 400 A.

Magnetoresistive sensors (MR) made from magnetic layers and based on anisotropic magnetoresistance (AMR) [6,7,8,9], giant magnetoresistance (GMR) [10,11,12,13,14,15], and the tunneling magnetoresistance effect (TMR) [16] are now extensively studied and used for detection of DC/AC currents.

The resistance behaviour of magnetic thin films (Fe, Co, Ni, or alloys like Permalloy—Ni_80_Fe_20_) is anisotropic (AMR effect) with respect to the applied field direction [6]. The alloy’s resistance depends on the angle between the magnetization and the direction of current flow. In a magnetic field, magnetization rotates toward the direction of the magnetic field and the rotation angle depends on the external field’s magnitude. The resistance changes roughly as the square of the cosine of the angle between the magnetization and the direction of current flow. Based on this effect and on the planar Hall effect (PHE) which appears in such structures as a consequence of the AMR effect [6], many sensing applications have been developed. Most of these sensors are obtained using integrated circuit technology [7,8,9], where the resistive elements are connected in a Wheatstone bridge configuration to get high detection sensitivity around 0 field and a better thermal stability of the output signal. The resistive elements have a large aspect ratio (about 10 nm thin, a few μm wide, and tens of μm long), such that magnetization naturally aligns over the longitudinal axis (easy axis of magnetization). The Barber Pole biasing technique [6,8,9] is used to linearize the transfer function. 

To achieve a uniform rotation of the magnetization in the resistive elements, the magnetic field must be applied parallel with the sensor’s surface and perpendicular to the easy axis of magnetization. In [7], eight AMR sensors (model KMZ51) were placed in a circular pattern around a conductor through which the current to be measured is flowing. A linearity error of ±0.05% in the current range of ±8 A, i.e., an absolute resolution of 4 mA was reported. In [8], the AMR sensors are placed above a U-shaped current trace, the system being encapsulated in a SOIC16 package type. 

Currents up to ±50 A can be measured with a zero offset current up to 120 mA. For a current range of ±5 A, the zero offset current can reach a maximum value of 60 mA; the sensitivity is 350 mV/A (with signal conditioning) with a non-linearity error up to 0.5% F.S. (full scale). In [9], the AMR chip with a Wheatstone bridge was placed above the U-shaped conductor. As a common factor, these sensors contain, in their structure, a compensation conductor located above the MR elements [8,9]. Through this conductor a feedback current is driven to compensate the external magnetic field so that the sensor always works around a single point. 

This feedback current is a measure of the detected current. Also, as the internal magnetization has no preferred direction along the longitudinal axis, a flipping of 180° can occur due to overcurrent spikes or due to exposure to certain external magnetic fields. This flipping of the magnetization results in a different sensitivity of the system. To overcome this problem, an internal coil (KMZ51) or external controlled magnetic field should be used to reset the magnetization to the initial orientation. Care should be taken to avoid a current passing directly underneath the device itself as the magnetic field generated by that current will be parallel to the printed circuit board (PCB) surface and will affect the functionality of the AMR sensors.

In 1988, the giant magnetoresistance (GMR) effect was discovered in a [Fe/Cr]_n_ magnetic multilayer. It was found that a change of relative magnetic moment orientation between adjacent magnetic layers results in a significant change of resistance. When the layers are magnetized in parallel, the resistance is at a minimum value, *R*_p_. When the magnetizations of the adjacent magnetic layers are antiparallel to each other, the resistance is at a maximum value, named *R*_ap_. The physical mechanism of the GMR effect is the spin dependent electric transport in ferromagnetic transition metals. Thus, a new and dynamic field in science, named spintronics, has emerged from this discovery. In 2007, the importance of this discovery was awarded with a Nobel Prize in Physics. Many different applications have been developed subsequently, including low field sensors, position sensors, velocity sensors, Magnetic Random-Access Memory (MRAM) [12], and hard disks read heads. GMR sensors offer high sensitivity, wide frequency range, small size, low power consumption, and they are compatible with many other state-of-the-art technologies [13]. GMR sensors also have a number of drawbacks, from which we can note nonlinearity, hysteresis, offset, and a temperature dependent output that can reduce measurement accuracy [14]. In addition, the output of some of GMR sensors is unipolar, which limits its application in AC measurements [2].

In terms of theoretical considerations, several methods have been proven effective in improving the GMR sensor response. Using a bias field parallel to the sensitive axis can shift the operating point of the sensor to the linear region, thus reducing the hysteresis behavior and creating a bipolar signal. This field can be created either by using a permanent magnet or a coil system with DC, AC, or short pulse currents which can have either open or closed-loop control [14]. Optimization in terms of signal measurement (such as using a differential measurement method) and acquisition can also be performed.

Regarding the application of GMR sensors as current sensors, a multitude of studies have been performed to improve their characteristics. In [14], a closed-loop operation was used to improve the linearity of the GMR sensor. Hysteresis modelling compensation is used in [11] to reduce hysteresis and temperature dependency. In [15], low frequency capture is used to extend the sensor response up to ±800 A. Compared with AMR sensors, GMR sensors, offer a higher sensitivity and, in most cases, are more stable to overcurrent or magnetic field spikes.

## 2. Materials and Methods

### 2.1. Principle of Operation

**Figure 1:**
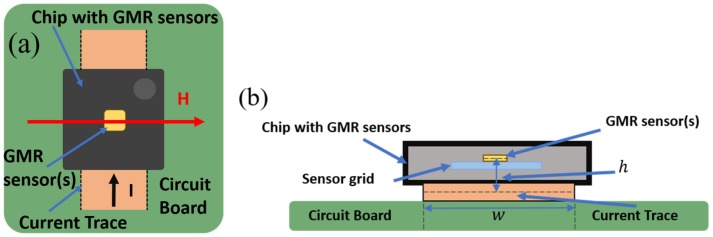
Non-contacting current measurement basic setup using a conducting trace and a giant magnetoresistance (GMR) based sensor chip: (**a**) plane view; (**b**) cross section (adapted from [17]).

The proposed current measurement method is an indirect one (the GMR sensor acts as a magnetometer by measuring the magnetic field produced by the current trace on which it is installed). Thus, if a current, I, passes through a wire, the magnetic field *B* will produce a change of the output voltage on the GMR sensor. Figure 1 illustrates the non-contacting current measurement demonstrator setup.

**Figure 2:**
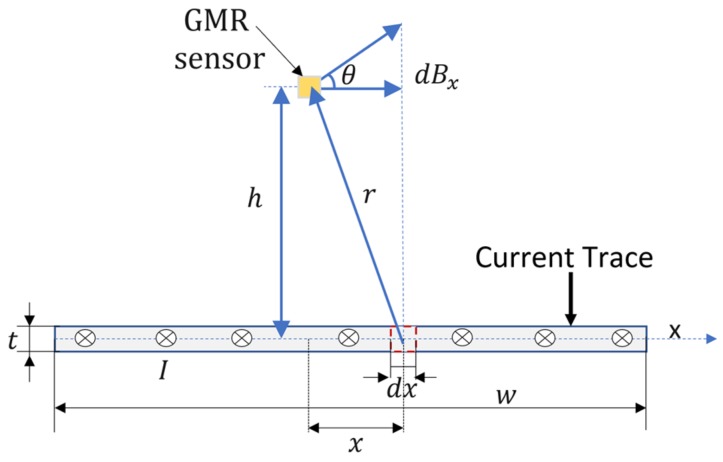
Cross section representing the parameters of the analytical model implemented for field calculations.

The current, *I*, from the conductive trace (denoted as “Current trace”) generates a magnetic field, whose component, *B_x_*, will be detected by the GMR sensor. To estimate *B_x_*, we derived an analytical method, which assumes that the sensor is centered above the trace at distance *h*, Figure 2.

Assuming a long conductive trace, the elementary field produced by the current *dI* is expressed, using the Biot-Savart law, by:

(1) $$dB=\mu_{0}\frac{dI}{2\pi r},dI=\frac{I}{w}dx$$

and

(2) $$dB_{x}=dB\cdot cos\theta =\mu_{0}\frac{h}{2\pi }\cdot \frac{I}{w}\cdot \frac{dx}{h^{2}+x^{2}}$$

where µ_0_ = 4π × 10^−7^ H/m is vacuum magnetic permeability; *w* is the trace width, and *h* is the distance from the trace to sensor.

Usually, the trace thickness is between 0.018 to 0.036 mm and *h* is about 0.4–0.8 mm for low-profile surface mount packages chips. So, we can assume, in Equation (1), a linear current density *I*/*w* to calculate the field.

By integrating Equation (2) and doing some basic calculations we obtain (where *I* is in A and *w*, *h* are in m):

(3) $$B_{x}=\left[\frac{I}{w}\cdot \arctan\left(\frac{w}{2h}\right)\right]\cdot 4\cdot 10^{-7}[T].$$

The results from Equation (3) can be expressed in [G] by:

(4) $$B_{x}=\left[\frac{I}{w}\cdot \arctan\left(\frac{w}{2h}\right)\right]\cdot 4\cdot 10^{-3}[G].$$

If h = 0.8 mm (for sensor AA003-02 produced by Nonvolatile Electronics (NVE) [13], *w* = 2 mm and I = 4 A, B_x_ = 7.16 × 10^−4^ T.

In the linear region of the sensor’s response we can express the output voltage as:

(5) $$\Delta U_{a}=S_{eff}\cdot B$$

where $S_{eff}$ is the effective sensitivity which depends on the sensor type and supply voltage.

For an AA003-02 GMR based sensor, *S* = 2.6 mV/(V × Oe). At a supply voltage *V_S_* = 5 V one obtains *S_eff_* = 13 mV/Oe and an estimated output voltage Δ*U_a_* = 93.18 mV if *I* = 4 A. The results obtained with this analytical method have proven to be consistent and more accurate than those that can be obtained by utilizing the web application from [17]. When the current in trace is smaller than 1 A, the magnetic field to be detected by a sensor becomes comparable with the earth’s magnetic field which implies some practical issues regarding low currents measurement.

### 2.2. Characterization of the GMR Sensor

The rate of change in the resistance of a GMR element is expressed by:

(6) $$GMR=\frac{R_{ap}-R_{p}}{R_{ap}}100\left[\%\right].$$

**Figure 3:**
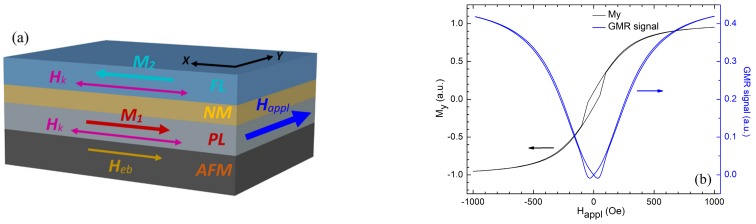
(**a**) Typical structure of a GMR sensor; (AFM—antiferromagnetic pinning layer; PL—pinned magnetic layer; NM—nonmagnetic spacer layer; FL—free magnetic layer (**b**) The simulated field dependence of the magnetization along the Oy axis (My) and the calculated GMR effect when *H_appl_* is directed over the Oy axis.

Usually, the multilayered structures, from which the GMR sensors are patterned, are of the type AFM/PL/NM/FL, Figure 3a, where AFM denotes an antiferromagnetic layer of IrMn, PL (named pinned layer or fixed layer) is a ferromagnetic layer of Ni_80_Fe_20_ (named Permalloy) or NiFeCo and NM is a very thin nonmagnetic layer of Cu (0.1–2 nm). The free layer, FL, also known as the sensing layer, as the magnetization can rotate upon an applied magnetic field, is usually deposited from Ni_80_Fe_20_ or NiFeCo. The GMR ratio for such structures is about 5%–15% [13,14]. The AA003-02 sensor, which contains two active GMR elements connected in a Wheatstone bridge, has a GMR ratio between 13%–16% [18]. 

Thus, a simple approach to simulate the field dependence of a GMR sensor signal is to calculate the behavior of the magnetization from the free layer (because the magnetization in the pinned layer can be assumed to be fixed for low applied fields.). For this purpose, we used the OOMMF (Object Oriented MicroMagnetic Framework) micromagnetic simulator [19]. The simulated layer is 1000 × 500 × 10 nm^3^ and consists from Permalloy; the cell size is 5 × 5 × 5 nm^3^. The FL is antiferromagnetically coupled with the PL through the NM layer, the coupling field being 200 Oe, along the Ox axis. The field, H_appl_, is applied perpendicular to the easy axis of magnetization (Ox), Figure 3a.

For simulations, we assumed M_s_ = 710 kA/m (saturation magnetization), A = 1.3 × 10^−11^ J/m (exchange constant), and an anisotropy constant, K_U_ = 804 J/m^3^ along Ox axis. These are typical material parameters used in micromagnetic simulations [19,20,21,22,23]. The cell size is determined by the exchange length, *l_ex_*, which for Permalloy is 5 nm [20]. To get reliable results, the side of the cell should not exceed *l_ex_*. However, sometimes, a larger cell size can be used if the simulation results converge to those obtained for 5 nm (or lower) and are in good agreement with experimental results. Also, care should be taken when reversal processes are studied, as we did in this paper, to show the hysteretic behavior of the magnetization along Oy axis and the GMR effect.

The saturation magnetization, Ms, can take values between 700 kA/m to 860 kA/m [19,20,21,22,23]. We found by VSM (vibrating sample magnetometer) measurements, on magnetic thin films with Permalloy (10 nm), that M_S_ = 710 kA/m, which is in agreement with [22], which shows a decrease of the saturation magnetization for very thin films. For the exchange constant, A, values between 10 pJ/m [20] to 13 pJ/m are reported [19,21,23]. We used A = 13 pJ/m. By using a larger value for K_U_ (instead of the default value of 500 J/m^3^) [19,23], we stressed the importance of the uniaxial anisotropy, typical for strips used to microfabricate GMR sensors, to keep the magnetization along the Ox axis when *H_appl_* = 0.

The simulated GMR response may be expressed as a function of relative magnetization angle, *θ*, between the free and pinned layer [23] with a relation of the type a+b(1−cosθ); a is a term which describes the structure resistance at saturation whereas *b* represents the magnitude of the GMR effect. For real structures, *a* and *b* depend on the stack structure. Figure 3b, presents the simulated field dependence of the magnetization along the Oy axis (My) and, based on this result, the calculated GMR response. The magnetic domain structure of the simulated layer is responsible for the small hysteretic behavior seen for the field dependence of My and GMR, even if the field is applied over the hard axis. In a single domain approach, there is no hysteresis for both My and GMR field dependencies, whereas in a multi domain approach, a hysteresis effect is present.

**Figure 4:**
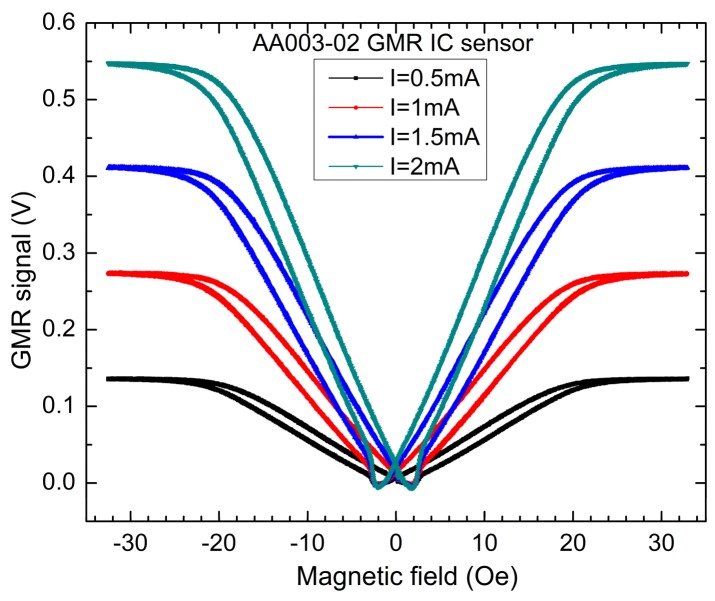
Typical measured field dependencies of the output signal for AA003-02 GMR sensor for different driving currents.

To sum up, the results presented in Figure 3b for the GMR effect are in good qualitative agreement with the data from Figure 4 which shows the typical measured field dependencies of the output voltage made on the AA003-02 sensor for different driving currents. One can observe that: (i) the sensitivity can be increased by supplying the sensors with a higher current (for example, I = 2 mA) and (ii) the sensor presents a nonlinear response around 0 field and low sensitivity around the coercive field. These observations motivate the necessity of a biasing field applied along the Oy axis. The driving current through the sensor was supplied by a Keithley 6221 source and the voltage was measured by using a Keithley 2812A nanovoltmeter. The magnetic field was generated by two rectangular-shaped coils in a quasi Helmholtz-like configuration which were supplied by a Kepco BOP100–10MG power supply.

### 2.3. GMR based Non-Contacting Current Sensing

**Figure 5:**
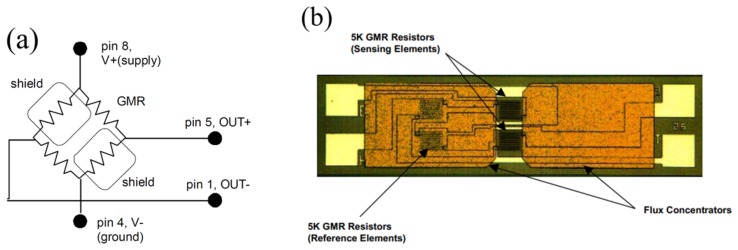
(**a**) NVE AA003-02E GMR sensor functional block diagram; (**b**) photomicrograph of an NVE sensor element [18].

Before implementing our differential measurement setup, initial tests were done using an evaluation kit, NVE AG003-01E, for current measurement [24] with AA003-02E GMR sensors. The AA003-02E sensor is a differential system on its own as can be seen in Figure 5a,b. The sensor operates as a Wheatstone bridge with four GMR elements, from which, two are magnetically shielded and two are active sensors. The structure is well balanced, such that it delivers an output voltage U~0 when H=0, as shown in Figure 4.

**Figure 6:**
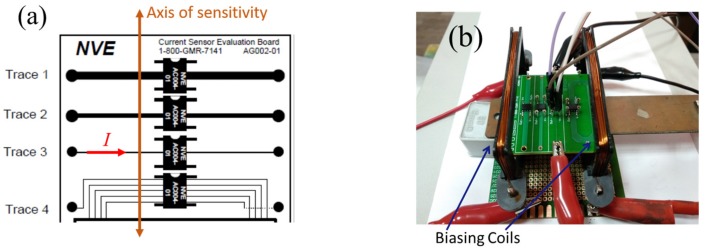
(**a**) Schematic of the evaluation board. The current trace 3 (w = 0.254 mm) was used for tests; (**b**) image of the experimental setup used for GMR current sensing.

A support holds the current sensor evaluation kit, Figure 6a, inside the coils, Figure 6b, which will be used to bias the GMR sensor in a linear region of its field-dependence characteristic, Figure 4.

**Figure 7:**
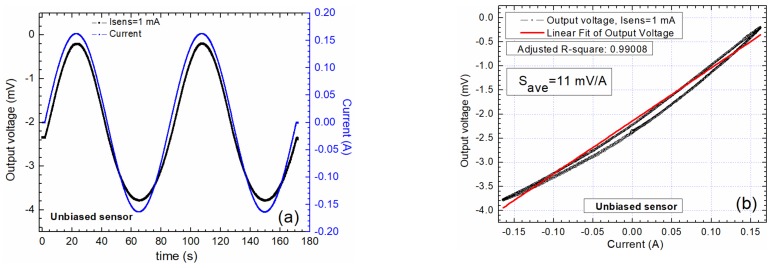
Output characteristics when the sensor AA003-02 is unbiased: (**a**) Comparison between the input current and the output voltage wave forms; (**b**) the output voltage as a function of the applied current through the current trace.

**Figure 8:**
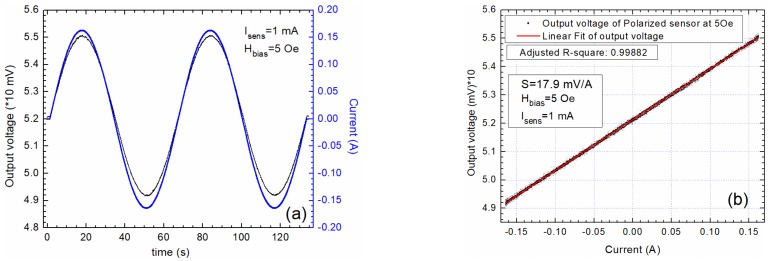
Output characteristics when the sensor AA003-02 is biased at 5 Oe: (**a**) Comparison between the input current and the output voltage wave forms; (**b**) the output voltage as a function of the applied current through the current trace; the sensitivity is 17.9 mV/A.

**Figure 9:**
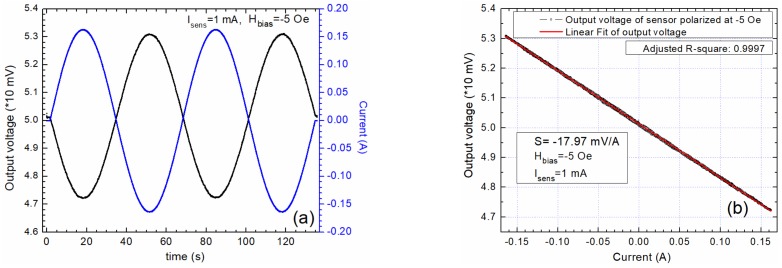
Output characteristics when the sensor AA003-02 is biased at −5 Oe: (**a**) Comparison between the input current and the output voltage wave forms; (**b**) the output voltage as a function of the applied current through the current trace; the sensitivity is −17.9 mV/A.

The detection system was characterized by applying a very low frequency 0.16 AC current in the conductive trace (Figure 7, Figure 8 and Figure 9). For these tests, the sensors were supplied with a constant current of 1 mA. The tests were made with unbiased (Figure 7), and biased sensors (Figure 8 and Figure 9). Figure 7 presents the output characteristics obtained when the sensor is unbiased. We must remark on the nonlinear response of the sensor and the hysteretic effect of the output signal, Figure 7a,b. 

When the sensor is biased at H_bias_ = 5 Oe or H_bias_ = –5 Oe, the output signals follow accurately the waveform of the applied current (Figure 8a and Figure 9a), and the sensor’s output is linearized with no hysteretic effects (Figure 8b and Figure 9b). Also, from Figure 8 and Figure 9, the importance of the biasing field polarity in relation with the polarity of the applied field (generated by the current I) is emphasized. That would allow an output signal in phase or out of phase with π with the applied current. These findings are used for designing the differential measurement setup in order to increase the sensitivity and to immunize the system from unwanted external magnetic fields and temperature fluctuations.

### 2.4. Differential Sensor Setup and Mode of Operation

**Figure 10:**
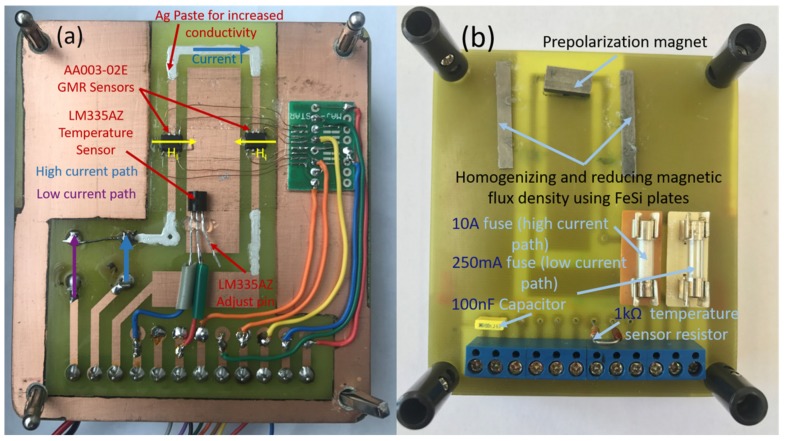
Custom PCB for current measurement using GMR sensors: (**a**) backside; (**b**) frontside. Note that the Ag paste is used to increase the cross section (and consequently, electrical conductivity) in the contacting areas, thus reducing the overall electrical resistance of the “U” shaped current trace.

A differential measurement system using two AA003-02E GMR sensors was developed. The PCB of the custom current measurement system can be seen in Figure 10. The GMR sensors are placed to operate in a differential configuration, i.e., for one sensor the output voltage increases while, for the second sensor the output voltage decreases when a current, I, is flowing through the U-shaped conductive band, Figure 10a. The width of the conductive band *w*, is 2 mm. In the same time, external magnetic fields, from unwanted sources are canceled using this setup. The high/low current path represent the same trace, the difference being the connected fuses used to protect the load during the tests. The 100 nF capacitor is used to filter the sensors supply voltage. Due to this mode of operation, it can be noted that the sensor is not affected by overcurrent because there is galvanic isolation between the sensors and the current trace. Even if the current produces a quite large magnetic field, this will not affect the sensor’s functionality, i.e., the magnetization of the pinned layer is not affected and the magnetization of the free layer will return to its initial orientation; this is because of the manufacturing technology [18] where an AF (antiferromagnetic) layer or a synthetic AF layer is used to bias the pinned layer. This means, there is no need of an external magnetic field to reset the sensors like is done in the case of many AMR sensors [7,25].

On the other hand, if we refer to overcurrent protection of the load, at this stage we did not implement the electronics used to trigger the protection when the corresponding signal from sensors surpasses a reference value.

Figure 10b shows the adjustable biasing system formed by a movable permanent magnet and two FeSi plates to homogenize (and also reduce) the effective magnetic flux density. In terms of design choices, the biasing field was set to 8 Oe. The system operates as follows: The permanent magnet generates a magnetic field in the direction of the sensitive axis of the GMR sensors (this shifts the GMR sensor response to a linear operation regime. Regarding the configuration of the permanent magnet, the magnetic field lines between the two Fe-Si plates are almost parallel, thus leading to a more homogeneous magnetic field at the location of the GMR sensors. This is done because the used magnet produces a much stronger magnetic field than is necessary for linearizing the sensors output, and can easily saturate the GMR sensor response for this kind of operation. The permanent magnet is precisely placed such that the polarization field for each sensor is almost the same. In order to increase/reduce this field, this magnet can be rotated or shifted up/down slightly when at the same time monitoring the sensors output to ensure similar polarizing fields.

**Figure 11:**
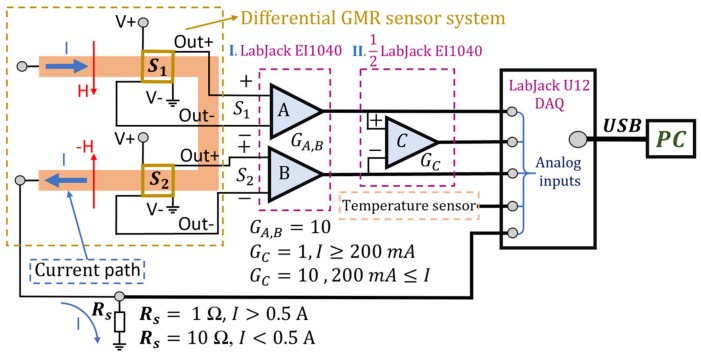
Current measurement differential system using GMR sensors: functional block diagram.

The functional block diagram of the experimental setup can be seen in Figure 11 and it consists of the custom PCB, LabJack EI1040 Dual Instrumentation amplifier [26], and a Labjack U12-DAQ card [27] connected to a PC via USB.

**Figure 12:**
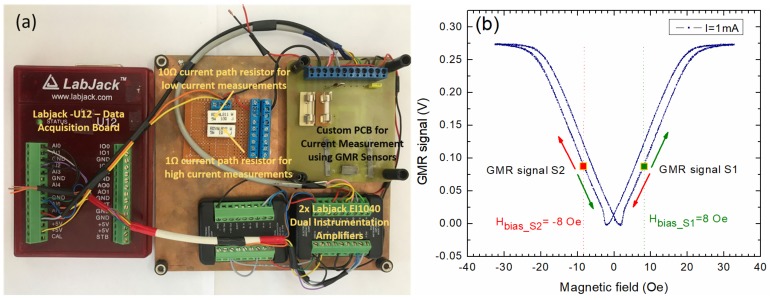
Differential measurement system: (**a**) experimental setup; (**b**) mode of operation illustration for H_bias_ = 8 Oe: when a current I is applied through the U-shaped band, the voltage on sensor 1 increases (green arrow) whereas the voltage on sensor 2 decreases (orange arrow). For H_bias_ = −8 Oe, the voltage on sensor 1 decreases whereas the voltage on sensor 2 increases when the same current I is applied (see Figure 8 and Figure 9).

In Figure 11, the amplifier setup for current measurement is also depicted. In this, case, a LabJack EI1040 Dual Instrumentation amplifier [26] is used to amplify the output signals from sensors; each channel was set to a gain of 10. The resulting signal is further amplified by another LabJack EI1040 amplifier which is set to a gain of 10 for low currents measurement, or 1 for high currents measurement. The resulting signals are sent to differential analog inputs on the LabJack U12 DAQ. Thus, for currents below 200 mA, the total resulting gain is 100. The gain for each instrumentation amplifier can be set manually or through the LabJack U12 digital input/output interface [27]. An image of the experimental setup can be seen in Figure 12a. For the purpose of this article, and practical implementation reasons, the AA003-02 GMR sensors were supplied with a 4.096 V constant voltage, generated by a thermally compensated source, from the EI 1040 Dual Instrumentation amplifier. For this voltage, the current through each sensor was about 0.8 mA (the internal resistance for each sensor is 5 kΩ as can be seen in Figure 5b). In order to avoid any possible contact with the current trace, the sensors were wired-bonded directly to the external circuit instead of mounting them on the PCB.

Since two almost identical AA003-02E sensors were used, the result is a double differential measurement system where the benefits and precision compared with a single differential measurement setup were further amplified. Figure 12b presents how the differential current measurement system operates: The sensors were both biased with a field of 8 Oe. From Figure 10a, one can note that since the sensors were placed in such a way that they operate in antiphase, the differential output from the sensors will subtract the influence of other external magnetic fields. In essence, any external homogenous magnetic field (not directly from the current trace) affecting both sensors equally will be subtracted from the differential output as one can see from Equation (7).

Although we agree that is impossible to have a measurement system totally immune to external magnetic fields, some specific properties of our differential system can be exploited to minimize these perturbations. As the sensors are made from very thin (nm) magnetic/nonmagnetic layers, they are not sensitive to perpendicular applied magnetic fields, lower than a few hundred Oe, due to the large shape anisotropy which keeps the magnetization in the film plane. Also, if the external magnetic fields are applied in the film plane but over a direction perpendicular to the axis of sensitivity, Figure 6a, the sensor’s response can be neglected for fields lower than 25 Oe (Figure 4).

Thus, we can note that the influence of the external currents can be minimized by a proper design of the measurement system using the following observations: The external current lines (if they exist in the sensor’s vicinity) must be directed parallel with the axis of sensitivity (i.e., the magnetic field they create is perpendicular to the axis of sensitivity). The differential configuration can be affected by non-homogeneous external magnetic fields but to meet such a situation, the system has to be in the vicinity of magnetic field sources like coils and ferromagnetic components that can induce distortions of the magnetic field lines. In such a situation, electromagnetic shielding must be applied to the detection system, Figure 10. Also, the effect of these perturbations can be minimized by digital signal processing.

Furthermore, resulting from the operation of the differential measurement system, the following general equation can be derived for an input parameter *x* and a temperature variation ΔT:

(7) $$y=(K_{S1}x+S_{1}\Delta T)-[K_{S2}\left(-x\right)+S_{2}\Delta T)]$$

where y represents the differential output, $K_{S1}$ and $K_{S2}$ are the sensitivities of each sensor for the useful input signal, and S_1,2_ΔT is the signal change caused by thermal fluctuations.

By taking into account that each sensor is thermally balanced, one can assume that S_1_ΔT→0 and S_2_ΔT→0. As the current through the trace creates a magnetic field $H_{I}=C\cdot I$ (where C is a constant) we can express the output voltage of the differential system as:

(8) $$\Delta U=(K_{S1}\cdot H_{I}+S_{1}\Delta T+K_{S1}\cdot H_{ext})-[K_{S2}(-H_{I})+S_{2}\Delta T+K_{S1}\cdot H_{ext}]$$

By rearranging the terms, Equation (8) becomes:

(9) $$\Delta U=(K_{S1}+K_{S2})\cdot H_{I}+\left(S_{1}-S_{2}\right)\cdot \Delta T+\left(K_{S1}-K_{S2}\right)\cdot H_{ext}.$$

By considering that *S*_1_≈*S*_2_ (for the same type of sensors), i.e., the system is thermally balanced, and the differences between the sensors output variation created by external fields are negligible, Equation (9) becomes:

(10) $$\Delta U=(K_{S1}+K_{S2})\cdot H_{I}=(K_{S1}+K_{S2})\cdot C\cdot I=S\cdot I$$

where *S* (V/A) is the sensitivity of the differential measurement system.

## 3. Results and Discussion

**Figure 13:**
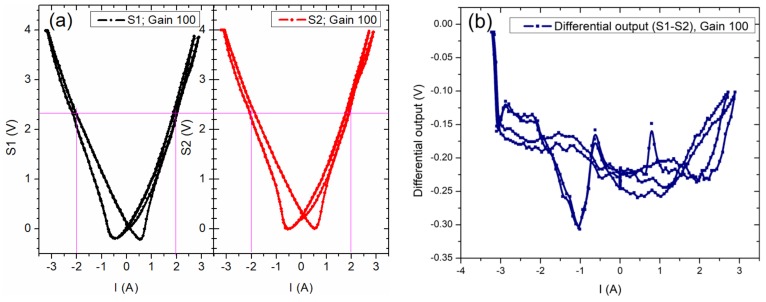
Measured signals on unbiased sensors for: (**a**) individual sensors; (**b**) differential setup. For these tests, the signal was amplified by 100 times.

The results presented in this section are a summary of many tests done for different input currents both in DC and AC. From Figure 13a we can denoted that the sensors response is nonlinear in the −1.5 A to 1.5 A current region, which would not allow low currents measurement without biasing. Figure 13b presents the output characteristic of the differential system obtained for unbiased sensors for a DC current between −3 A to 3 A. The response from each sensor is slightly different and presents a hysteretic behavior. The differential output is chaotic, and thus unusable.

**Figure 14:**
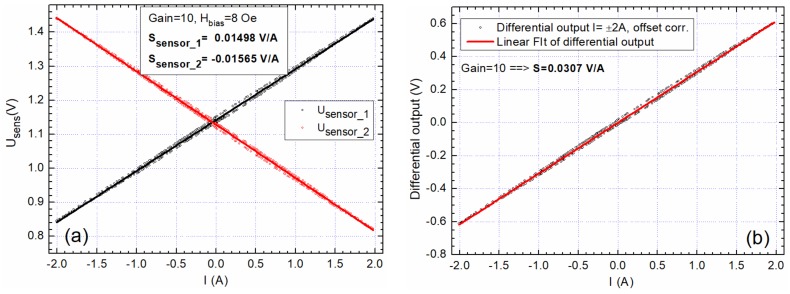
Differential output of sensors polarized at 8 Oe, DC ±2 A: (**a**) individual sensors response; (**b**) differential output.

In what follows, the results obtained with sensors biased at 8 Oe and using the setup from Figure 10 and Figure 11 will be presented. Figure 14 presents the system response when measuring a variable DC current between −2 A to 2 A. The sensitivity for the differential output is *S* = 0.0307 V/A. Due to inherent hysteresis effects (note Figure 12b and Figure 13a), a hysteresis effect of 0.04 A was observed in the range of ±2 A.

**Figure 15:**
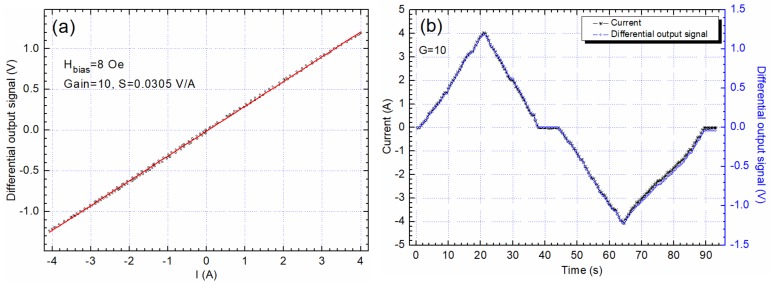
The response of the differential system when the current varies between −4 to 4 A following an arbitrary wave form: (**a**) differential output characteristic, (**b**) the signals variation over time.

In Figure 15a, the system’s output when measuring a variable DC current from −4 A to 4 A is presented, while Figure 15b presents the signals variation over time. As expected, the sensitivity is almost the same but the hysteretic effects are lower. Above 4 A, the thermal stability of the setup is negatively impacted as heating occurs.

For the differential measurement system, the temperature drift of the offset can, theoretically, go to zero for sensors that perfectly matched and are subjected to the same biasing field. The temperature drift of the offset was measured with the sensors biased in order to place them in a linear operation regime and to have the same (almost) output voltage when no current is applied in the conductive band, Figure 12b. The measured temperature drift of the offset is ΔU_0_/ΔT ≈ −7.9 × 10^−6^ V/°C which means about −2.59 × 10^−4^ A/°C in terms of measured current, for a temperature variation of 20 °C. Thus, it can be noted that the temperature drift of the offset is affected mainly by the temperature dependence of the GMR effect. Also, we can note that any temperature drifts in the operating range of the bias magnet and FeSi plates lead to no significant changes to the bias magnetic field as we estimate that the temperature of these components is no larger than 37 °C during our tests. Moreover, we used a ferrite magnet from NVE to bias the sensors (which has a Curie temperature up to 300 °C).

**Figure 16:**
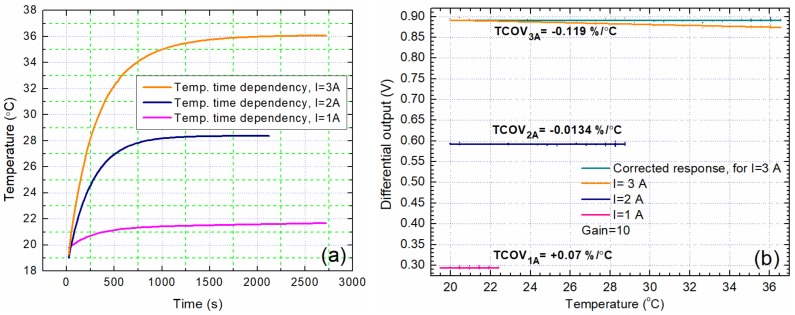
(**a**) The time dependency of the sensors temperature for I = 1 A, 2 A, and 3 A; (**b**) the thermal drift of the differential output. The temperature variation is caused by the Joule heating of the conductive band.

The thermal drift of the sensor is defined by the TCoutput change with temperature using a constant current source) and TCOV (output change with temperature using a constant voltage source). According to the catalogue [18], for a single sensor, TCIO is +0.03 %/°C, while TCOV is −0.1 %/°C. Since the sensors are supplied with 4.096 V constant voltage, TCOV is relevant in this case. An LM335AZ temperature sensor was mounted on the PCB for measuring temperature (Figure 10a). Figure 16a shows the time dependence of the temperature of the PCB in the sensors vicinity for I = 1 A, 2 A, and 3 A respectively. One can observe that for a current of 3A passing through the conductive band, the temperature reaches a plateau at about 36 °C after 2000 s. Figure 16b shows the thermal drift of the differential output for I = 1 A, 2 A, and 3 A. The obtained values are: TCOV_1A_ = 0.07 %/°C, TCOV_2A_ = −0.0134 %/°C, and TCOV_3A_ = −0.12 %/°C. 

We can identify two possible effects responsible for the measured thermal drifts: (i) variation of the resistance of the metallic layers with temperature and (ii) temperature dependence of the GMR effect. The influence of the first effect is almost canceled by the Wheatstone bridge connection of the sensors inside the chip, Figure 5a, and by the differential measurement setup, Figure 11. This can be seen from data presented in Figure 16b, when very low magnetic field is applied to sensors for I = 1 A and 2 A respectively. On the other hand, the effect of spin fluctuations is shown to play an important role in the temperature-dependency of the GMR amplitude. As a consequence, the GMR effect shows an almost linear decrease when temperature is raised [28,29].

From Figure 16b we found a linear decrease of the output voltage, which is more important for I = 3 A where a larger amount of heat can be transferred to sensors and, hence, we expect a larger temperature variation of the GMR effect. This has an effect on the setup we used, but this can be compensated by applying a correction factor proportional with the measured temperature variation and using the calculated TCOV. Figure 16b presents the compensated response for I = 3 A through the conductive band.

Thus, we can note that the system is thermally stable and can provide reliable data within a temperature interval between 20 to 37 °C.

**Figure 17:**
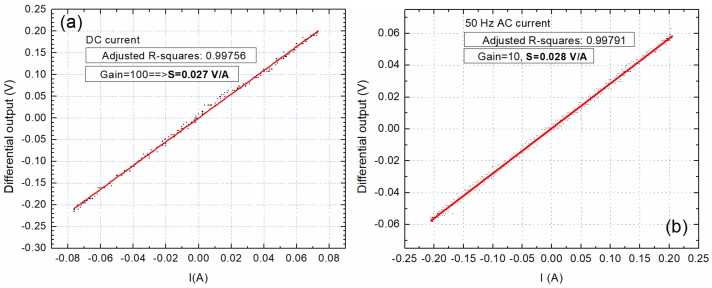
Differential output of sensors polarized at 8 Oe: (**a**) DC, 75 mA, (**b**) AC, 200 mA.

In terms of low currents sensing capabilities, the limitation is due to some factors like: the sensor’s field sensitivity, electric noise of the detection setup, and the width of the current path. We found that the implemented differential system is effective with currents as low as 75 mA, Figure 17a. For lower currents, the signal from the sensors is very weak and more precautions should be taken into account regarding electrical shielding, the noise of the signal amplifier, and the DAQ system. As we can see from Equation (3) and Figure 8 and Figure 9, a current line with a smaller width favours the measurement of low currents. However, a larger width of the conducting band is needed for measuring larger currents without excessive heating. For example, when I = 75 mA and w = 0.254 mm, H = 0.186 Oe, whereas H = 0.1344 Oe for I = 75 mA and w = 2 mm respectively. 

In Figure 17b, the response of the system when measuring a 200 mA, 50 Hz, alternative current is shown. In this case, a current of 150 mA is required in order for the output to be sufficiently linear. Below these thresholds, the nonlinearities in the sensor’s response provide an inaccurate differential output. That is due to the fact that at low currents, the sensors output no longer accurately follows the waveform of the magnetic field generated by said current. Thus, the output signal appears distorted and does not represent the actual sine waveform. This is also true when measuring DC currents, as the differential output can be scattered creating some nonlinearities in the response (Figure 17a).

**Figure 18:**
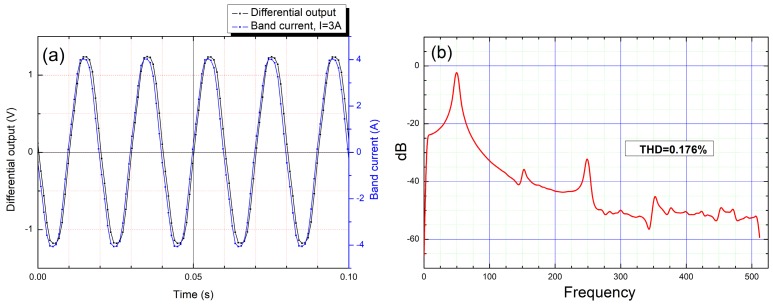
Biased sensors, AC 3A: (**a**) differential response and band current; (**b**) harmonic analysis.

In Figure 18a, the AC response of the system when measuring a 50 Hz sine waveform at 3 A is shown. The harmonic analysis for this measurement is shown in Figure 18b. A THD (total harmonic distortion) of 0.176% was obtained in this case. We can notice that the signal integrity is very good with little to no distortion (the fundamental frequency is the major amplitude, while the effect of the 3rd, 5th, and 7th harmonics is negligible). Note that the frequency limits of the response in AC are mostly limited by the DAQ system, as the sensors have a theoretical maximum frequency response of 1 MHz [18]. Further studies can be done to find the actual AC frequency limitations of the system. 

**Figure 19:**
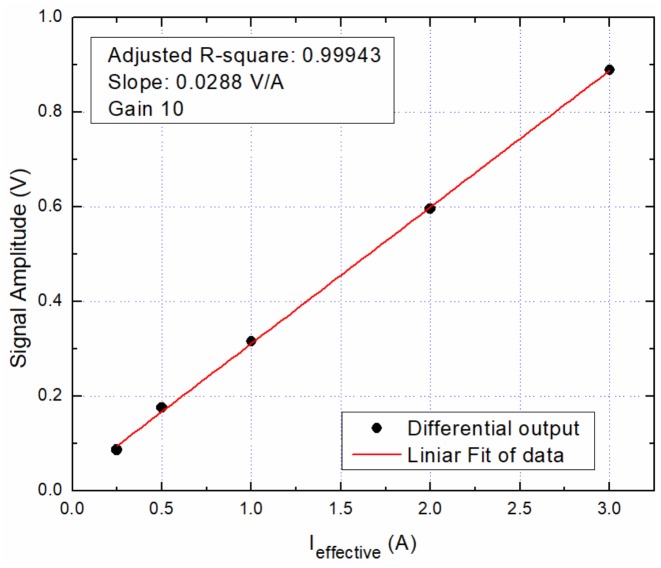
8 Oe biased sensors: AC calibration curve within the 0–3 A range.

Figure 19 shows the AC calibration curve for the device within the 0–3 A range. We used the adjusted R-squared term to show how well data is aligned over the fitting line. The adjusted. R-square is 0.99943. The calculated full-scale error is 0.66%. Note that there is a very good correlation between the measured current and the response of the system.

What is noteworthy for the implemented system’s output is that all the signal acquisition is done without implementing any filtering system. In this way, the system’s viability to measure both DC and AC currents was demonstrated. Thus, it can be noted that for a specific application (in DC or AC), further signal improvements can be made.

## 4. Conclusions

A high sensitivity non-contacting current measurement experimental setup based on giant magnetoresistance (GMR) sensors was implemented. The sensitivity of this detection setup is between 0.0272 to 0.0307 V/A with low (40 mA) hysteretic effects. A biasing magnetic field was used to linearize the field dependences of the sensors. Moreover, the implemented differential GMR system is very versatile, being able to measure both DC and AC currents. The current measurement system (Figure 12a) was proven to be able to measure accurately and for extended periods of time in DC from 75 mA up to around 4 A, and in AC from 150 mA up to 4 A. This system has the following advantages: high sensitivity, galvanic isolation, thermal stability (when operating at specified parameters), immunity to low external magnetic fields, and preservation of signal integrity for the input current, as can be seen in Figure 15 and Figure 18. These results were obtained without EMF shielding or filtering systems. The custom PCB for the system was designed to measure currents up to 10 A (by taking into account the copper trace width [17]), however, in practice, it was observed that significant heating occurs when measuring currents larger than 4 A for an extended period of time (Figure 16).

Moreover, in terms of performance comparison of the implemented sensor setup with other solutions on the market, we can note the following: The novelty of our approach consists in using a double differential measurement system, Figure 11, based on commercial GMR sensors, with an adjustable biasing system used to linearize the field response of the system. This approach was not seen in other works [14,30,31,32] or was implemented in commercial sensors like microfluxgate [4,5] or based on AMR effect [7,8,9]. As we are using a movable permanent magnet to bias the sensors and there is no compensation coil, the power consumption of our detection system (DAQ card and PC is not included) is very small, of about 6.4 mW (as each sensor has a power consumption of 3.2 mW, as noted in [18]). 

To improve the measurement accuracy of a magnetometer using the same type of sensor like we used in this study, a closed-loop GMR–compensation coil is used in [14,30], the system operating similarly as in [8,9]. With this method, a sensitivity of about 0.03 V/A to 0.04 V/A (with signal conditioning) is reported in [30] which is quite similar to our result obtained without a feedback coil. The power consumption was reported to be 1.6 W at low currents through the conductive band to 3.2 W for currents up to 45 A. 

In [9], for the MCA1101-xx-5 series current sensors, a sensitivity between 35 mV/A up to 350 mV/A for current sensors in the 5–50A range which is typical for AMR effect sensors, but lower than GMR based sensors. In [31], a temperature coefficient TCOV of −0.17 %/°C of the sensor’s output voltage is obtained while for our system a TCOV between −0.0134 %/°C to −0.117 %/°C has been measured. Also, in [8], typical CMS2000 series AMR sensors, have a typical offset voltage at room temperature of ±20 mV compared with our setup of −7.9 × 10^−6^ V/°C. This result emphasizes the benefit of our double differential measurement system to lower the thermal drift of the output signal.

Furthermore, the present setup aims to serve as a novel proof concept of concept application, and with future development, the operation range and utility of the system can be improved greatly. The current implementation is a compromise between low current and high current measurement. For example, by taking into account, Equation (4), we can note that low currents sensing capabilities can be improved by using a narrower trace. Also, for high currents measurement, a setup utilizing a much thicker trace and thicker PCB can be used. Thus, by redesigning of the setup, a significant increase in the operation range can be achieved. Further improvements can also include a size reduction (by integrating the amplifiers on the same PCB), EMF shielding and implementing a filtering system.

Finally, the differential sensing method presented in this article can be used for other specific applications requiring a high degree of sensitivity. As measuring low currents implies accurate detection of magnetic fields smaller than 0.5 G, some of the results presented in this paper will be used to develop a high sensitivity detection setup of magnetic nanoparticles (MNPs) used to label biomolecules in lab-on-a-chip (LOC) applications [33,34,35,36,37]. As we showed by micromagnetic simulations [35,36] and experiments [34], to achieve a large signal from MNPs, they must be polarized in quite a large magnetic field that can saturate the spintronic sensors. To avoid this, we proposed a specific polarization setup for MNPs, where the field is applied perpendicular to the sensor’s surface [35,36,37]. The MNPs will be localized on the surface of one GMR sensor whereas the second one will be used as reference sensor. The in-plane components of the magnetic fields locally generated by MNPs will be detected by GMR sensors using the differential setup described in Figure 11. The current through the conductive band will be used to produce an AC excitation field for detection of the MNPs. To ensure a smaller distance between MNPs and GMR sensors, a package flip-chip package type will be used in this development, as in [34].
